# Exploring the combined effects of sleep apnea and APOE-e4 on biomarkers of Alzheimer’s disease

**DOI:** 10.3389/fnagi.2022.1017521

**Published:** 2023-01-04

**Authors:** Arlener D. Turner, Clarence E. Locklear, Daisha Oruru, Anthony Q. Briggs, Omonigho M. Bubu, Azizi Seixas

**Affiliations:** ^1^Center for Translational Sleep and Circadian Sciences, Department of Psychiatry & Behavioral Sciences, Miller School of Medicine, University of Miami, Miami, FL, United States; ^2^Department of Neurology, Grossman School of Medicine, New York University, New York, NY, United States; ^3^Department of Psychiatry, Grossman School of Medicine, New York University, New York, NY, United States; ^4^Department of Population Health, Grossman School of Medicine, New York University, New York, NY, United States; ^5^The Media & Innovation Lab, Department of Psychiatry and Behavioral Sciences, Miller School of Medicine, University of Miami, Miami, FL, United States

**Keywords:** Alzheimer’s disease, sleep apnea, APOE-e4, amyloid, biomarkers, Black/African American population

## Abstract

**Objective:**

We determined the interactive associations of apolipoprotein e4 (APOE-e4), and obstructive sleep apnea (OSA) on biomarkers of Alzheimer’s disease and examined for racial/ethnic differences of this association.

**Methods:**

We used data from the National Alzheimer’s Coordinating Center Uniform Dataset (NACC UDS). All participants undergo annual observations, including demographic survey, battery of neuropsychological tests, blood draw (with genotyping), and a clinical evaluation with medical and cognitive/dementia status assessment, while a subset of participants have cerebrospinal fluid (CSF) biomarkers and neuroimaging data. Biomarkers of AD were characterized as the presence of abnormally low amyloid in CSF, *via* validated Aβ_42_ cut off protocols, and total segmented hippocampal volume, and volume of white matter hyper intensities (WMH). While clinical markers (to preview cognitive relationships) were characterized *via* the Montreal Cognitive Assessment (MOCA).

**Results:**

Biomarker and clinical marker data were derived from 1,387 participants at baseline (mean age = 69.73 ± 8.32; 58.6% female; 13.7% Black/African American), 18.4% of the sample had sleep apnea, and 37.9% were APOE-e4 carriers. Our results confirmed previous reports that OSA and APOE-e4 were independently associated with AD through abnormal levels of amyloid (*F*_(1,306)_ = 4.27; *p* = 0.040; *F*_(1,285)_ = 60.88; *p* < 0.000, respectively), WMH volume (*F*_(1,306)_ = 4.27; *p* = 0.040; *F*_(1,285)_ = 60.88; *p* < 0.000, respectively), and MOCA scores (*F*_(1,306)_ = 4.27; *p* = 0.040; *F*_(1,285)_ = 60.88; *p* < 0.000, respectively). No significant interaction between OSA and APOE-e4 relative to amyloid emerged, however, race stratified analyses indicated the interaction of OSA and APOE-e4 and was significantly associated with WMH and hippocampal volume in Black/African American, but not white participants.

**Conclusion:**

OSA and APOE-e4 are interactively associated with WHM in Black/African Americans. This interaction may partially explicate increased levels of risk in this population.

## Introduction

Alzheimer’s disease (AD) is the most common cause of dementia in the United States, impacting approximately 6.5 million individuals over the age of 65, a number that is slated to increase to 12.7 million by the year 2050 (Anonymous, 2022). The pathological course of AD includes the intracellular deposition of phosphorylated tau, extracellular accumulation, and deposition of amyloid beta (Aβ) and structural brain atrophy (hippocampus)(Brayne et al., 2009; Jack et al., 2018) research generally focuses on the biomarkers of AD (PET imaging, CSF, and/or plasma levels of Aβ and tau, MRI of hippocampal atrophy), to predict the future development of the disease. However, the etiology of AD is multifaceted and heterogeneous with numerous contributors to its pathophysiology (Olsson et al., 2016; Schindler et al., 2019).

Sleep disturbances, and specifically obstructive sleep apnea (OSA), which is characterized by reductions in and cessation of breathing during sleep leading to hypoxia and sleep fragmentation (Daulatzai, 2015), has been recognized as a factor that increases risk for, manifestations of, and possibly progression of AD (Andrade et al., 2018; Shi et al., 2018; Lal et al., 2022) Several mechanisms of OSA, have been indicated to contribute to cognitive impairments, neurodegeneration, and an increased risk of developing AD (Fernandes et al., 2021). There are two well documented mechanistic pathways of OSA leading to AD. One mechanistic pathway is through an exacerbation of the vascular hypothesis which suggests that hypoxia inherent in OSA causes oxidative stress and neuroinflammation increasing cell death and amyloid accumulation (Daulatzai, 2015; Scheffer et al., 2021). The second mechanistic pathway is through sleep fragmentation reducing slow wave sleep, in turn impedes glymphatic flow, and therefore the delicate interplay between amyloid production and clearance (Xie et al., 2013; Rainey-Smith et al., 2018). The idea of these mechanistic pathways is backed by the fact that OSA has been associated with higher levels of Aβ in both cerebrospinal fluid (CSF) and PET imaging, as well as faster annual increases in CSF and PET Aβ, and faster annual decreases in CSF Aβ42 levels, in both mild cognitive impairment and cognitively normal samples (Ju et al., 2016; Liguori et al., 2017; Elias et al., 2018; Bubu et al., 2019, 2020). Additionally, the apolipoprotein e-4 allele (APOE-e4), found in approximately 20% of the American population, and associated with a significantly increased risk for AD, has also been reportedly related to sleep disordered breathing. The e4 variant of APOE, is the largest known genetic risk factor for AD in a variety of ethnic groups and is also reported to be associated with the progression and severity of OSA, and cognitive dysfunction (Roses, 1996; Tardiff et al., 1997; Kadotani et al., 2001; Gottlieb et al., 2004; Conejero-Goldberg et al., 2014; Uyrum et al., 2015; Spira et al., 2017; Burke et al., 2018).

It has been hypothesized that vulnerability to cognitive deficits is increased in carriers of the APOE-e4 allele due to a limited response to physiological challenges (O'Hara et al., 1998; Johnson et al., 2017), and that OSA may potentiate neuroinflammatory processes associated with APOE-e4 by augmenting inflammation through hypoxia (O'Hara et al., 1998; Dewan et al., 2015; Johnson et al., 2017). Though it is now considered an accepted framework that a combination of genetic and environmental factors explains individual predisposition for AD development the specific underlying pathophysiological mechanisms are still elusive. Age and genetic background, (the presence of the APOE-e4 genotype) are important non-modifiable risk factors for AD (Rajan et al., 2017; Anonymous, 2021). While APOE-e4 has been linked to a 2- to 3-fold increased risk of AD in heterozygotes (i.e., e2/e4 or e3/e4 genotypes) and 10 times the risk in homozygotes (ε4/ε4), compared to carriers of the most common APOE genotype, homozygotes of e3 (ε3/ε3) (Corder et al., 1993; Spira et al., 2017). While 20–30% of the population has one or more e4 alleles, which has been shown to place them at elevated AD risk (Evans et al., 2003; Blair et al., 2005; Beydoun et al., 2013; Spira et al., 2017), it is important to note there is evidence that the APOE genotype does not always predict AD among individuals who identify as Black/African American ^(^Evans et al., 2003; Blair et al., 2005; Beydoun et al., 2013; Qian et al., 2017; Rajan et al., 2017; Spira et al., 2017; Weiss et al., 2021) Given that evidence has proposed that increased risk related to the social/cultural construct of race/ethnicity may stem from disparities in health conditions, socioeconomics, and life experiences, secondary to systemic racism (Rajan et al., 2017; Anonymous, 2021) it is imperative to explore environmental influences related to these racial differences in APOE genotypical risk.

Though OSA and the APOE-e4 allele have been analyzed separately and found to be predictors of AD, the relationship or potential interaction between the two predictors has not been extensively explored and is not fully understood. Moreover, these factors have not been examined in relation to racial differences. It is possible that racial differences in risk incurred from APOE-e4 stem from a paucity of explorations into the interaction between genetic and environmental factors. Therefore, we utilized baseline data from the National Alzheimer’s Coordinating Center (NACC) to explore the combined effect of OSA and APOE-e4 on biomarkers and clinical phenotypes of AD, as well as, whether these associations differed in Black/African American and White samples.

## Materials and methods

We utilized the National Alzheimer’s Coordinating Center (NACC) Uniform Data Set (UDS) to examine the effects of both static and modifiable risk factors (through the presence of OSA and APOE-e4, respectively), on biomarkers and phenotypical presentations of AD, and to explore whether the presence of sleep apnea conferred greater risk on older Black/African Americans specifically. The NACC UDS is a longitudinal database of standardized clinical, neuropsychological, and neuropathological research data started in 2005, using data collected from NIA-funded Alzheimer’s Disease Research Centers (ADRCs) across the United States (Beekly et al., 2004). The current study utilized data collected between September 2005 and March 2022 and includes data from 21 ADRCs in the analyses. Though the NACC database itself is exempt from IRB review and approval because it does not involve human subjects, as defined by federal and state regulations, all contributing ADRCs obtain informed consent from their participants and maintain their own separate IRB review and approval from their institution prior to submitting data to NACC. Therefore, all procedures performed in this study involving human participants were completed in accordance with IRB standards and with the Helsinki declaration and its amendments.

The participants’ first visit was considered as the baseline data. Participants voluntarily presented to one of the ADRCs for approximately annual observations, including a full battery of memory and non-memory neuropsychological tests, and a clinical evaluation. Versions 2 and 3 of the UDS neuropsychological battery were used by ADRCs for assessment and diagnosis during the time period for which the current data was collected, however, due to the data points examined, all included neuropsychological tests are from Version 3. The full neuropsychological battery has been described extensively in the literature (Beekly et al., 2004; Monsell et al., 2016). Participants could also elect to participate in additional study aims by undergoing neuroimaging, providing blood, CSF, and other specimens for genotyping and analysis.

The analytic sample includes individuals with no missing demographic data who identify as either Black/African American or White, and also have no missing data for the presence of sleep apnea, or APOE-e4 genotyping (*n* = 1,387).

### Outcome measures

The presence of sleep apnea at the first visit was assessed *via* self-report to the clinician during the clinical evaluation/interview and is regarded as being either present or absent (1 or 0 respectively).Sleep apnea and other assessments of sleep behavior were introduced in UDS version 3 (implemented in March 2015), which was likely the reason for the included data only utilizing Version 3 when Version 2 was also available, as well as the relatively large volume of missing data related to sleep apnea. It is important to note that given the recency of sleep data collection the NACC UDS repository lacks objective sleep measures like polysomnography or actigraphy data.

APOE genotype was determined independently by the ADRC, using either a buccal swab or blood draw. All six possible genotypes were reported to NACC. Individuals with APOE e2/e4, e3/e4, and e4/e4 were considered e4 carriers and individuals with APOE e2/e2, e3/e3, and e2/e3, were not e4 carriers. For the purposes of this study, we considered not just whether the participant was an APOE-e4 carrier or not, but focused on dose level, i.e., zero, one, or two, alleles present.

The biomarkers explored for theses analyses include abnormal levels of Aβ, hippocampal volume, and white matter hyperintensity (WMH) volume, while the clinical markers explored include cognitive functioning *via* neuropsychological performance and cognitive/dementia status.

Experienced clinicians classified participants with respect to dementia according to criteria of the joint working group of the National Institute of Neurological and Communicative Disorders and Stroke and the Alzheimer’s Disease and Related Disorders Association (McKhann et al., 2011). Data utilized for these classifications include (but are not limited to), performance on version 3 of the uniform data set neuropsychological battery. The full neuropsychological battery has been described elsewhere (Beekly et al., 2004; Monsell et al., 2016). The battery includes a measure of global cognition *via* the Montreal Cognitive Assessment (MOCA), which is used in these analyses to preview the relationship with cognitive functioning.

Given the nature of amyloid measurement across ADRCs, all available assessments of amyloid measured *via* CSF were assessed. The dataset returned two such measures, presence of abnormally low amyloid in CSF, a dichotomous variable, and levels of Aβ in CSF in pg./ml, a continuous variable. The majority of participants with reported amyloid had data for one of these variables and not the other, therefore we created a variable for abnormal amyloid levels that combined results from both NACC presented variables. We dichotomized the continuous variable such that any pg./ml Aβ level below the CSF Aβ_42_ cut off suggested by the NIA-AA/ADNI/Knight ADRC protocols for the measurement type (i.e., ELISA or Luminex) (Fagan et al., 2007; Jack et al., 2018) were rated as yes for abnormal amyloid, while levels above the cut off were rated no. The abnormal amyloid levels variable represents a 1 (yes) or 0 (no) for individuals who had the same score for either the abnormally low amyloid variable or the newly dichotomized pg./ml Aβ level variable. Twenty participants had data for both variables and all 20 scores were in agreement regarding the presence or absence of abnormal Aβ levels, encouraging security in the new variable.

The NACC neuroimaging database contains a sample of MRIs and PET scans that are linked to the standardized clinical and neuropsychological data and can also be linked to genotype data. For analytic purposes, this study utilized the calculated summary data for imaging parameters as presented within the NACC dataset. The calculations for the calculated summary data were performed by the IDeA Lab (Director: Charles DeCarli, MD; University of California, Davis^1^), following ADNI protocols. Hippocampal volume was characterized in each ADRC center from cortical reconstruction and volumetric segmentation performed with Freesurfer image analysis suite, a well-documented and freely available online software. Hippocampal volume data obtained from NACC represents the segmented total hippocampi volume as calculated per the EADC-ADNI harmonized protocol. White matter hyperintensity volume obtained from NACC represents the volume of white matter hyperintensities as delineated by calculated summary data provide by the IDeA Lab.

### Data analysis

For descriptive purposes characteristic variables were compared using chi-square and t-tests for categorical and continuous measures, respectively.

This study utilized a series of logistic regression and two-way analysis of variance (ANOVA) analyses to explore the associations among presence of sleep apnea and number of APOE-e4 alleles (zero, one, or two) on biomarkers (abnormal levels of Aβ, hippocampal volume, and WMH volume), and clinical markers (cognitive functioning)of AD. Post-hoc analysis was performed using Simple & Repeated contrasts, and Tukey’s multiple comparison test to compare differences between the number of APOE-e4 alleles a participant possessed for each significant variable of interest.

We also utilized the Regression and ANOVA analyses, along with subsequent analyses grouping the dataset into Black/African American and White cohorts to examine the effect of any variable interactions in these groups as well as account for any effects of race and sex. All analyses were performed using the Statistical Package for Social Sciences (IBM, 2021) and a *p* < 0.05 was considered statistically significant.

## Results

### Descriptive results

Analyses were conducted on the 1,387 participants 1,197 self-identified as white, and 190 self-identified as Black/African American), with both APOE-e4 and Sleep Apnea data. The mean age of the total sample was 69.73 (SD 8.32). Within the total analytic sample 58.6% were female, 86.3% were White, and 13.7% were Black/African American. Most participants (43.8%) were college graduates or had education beyond college (42.6), and a few (13.3%) had high school or lower education. Approximately 37.9% were APOE-e4 carriers and 18.4% had sleep apnea. Table 1 demonstrates these characteristics in White and Black/African American participant cohorts and displays percentages, means, and standard deviations (where applicable) including details on frequency of APOE genotype and degree of e-4 allele. Additionally, Table 2 depicts the sample characteristics by presence of Sleep Apnea.

**Table 1:** Sample characteristics.

	Total sample (*n*=1197)		Black participants (*n*=190)		White participants (*n*=1197)		*F /t/X^2^*	Sig
Age *mean (SD)*	69.7	(8.3)	68.5	(8.4)	69.93	(8.3)	**2.21**	**0.014**
Female sex	813.0	58.6%	140.0	73.7%	673	56.2%	**20.61**	**<0.001**
*Education*								
Years *mean (SD)*	16.1	(2.8)	16.2	(2.8)	15.47	(2.8)	**3.21**	**<0.001**
<9 | Elementary School	19.0	1.4%	1.0	0.5%	18.0	1.5%	**14.81**	**0.002**
9-12 | High School	165.0	11.9%	38.0	20.0%*	127.0	10.7%*		
13-16 | College	607.0	43.9%	80.0	42.1%	527.0	44.2%		
>17 | Graduate School	591.0	42.0%	71.0	43.6%	520.0	43.6%		
*Cognitive Status*								
Normal Cognitive Status	671.0	48.4%	106.0	55.8%*	565.0	47.2%*	**11.16**	**0.011**
Impaired, not MCI	51.0	3.7%	11.0	5.8%	40.0	3.3%		
MCI	450.0	32.4%	55.0	28.9%	395.0	33.0%		
Dementia	215.0	15.5%	18.0	9.5%*	197.0	16.5%*		
*APOE-e4*								
APOE e4 Carrier	521.0	37.9%	80.0	42.1%	446.0	37.3%	1.64	0.116
Frequency of alleles							**26.38**	**<0.001**
e3/e3	719.0	51.8%	76.0	40.0%*	643.0	53.7%*		
e3/e4	406.0	29.5%	70.0	36.8%*	339.0	28.3%*		
e3/e2	135.0	9.7%	32.0	16.8%*	103.0	8.6%*		
e2/e4	28.0	2.0%	4.0	2.1%	24.0	2.0%		
e4/e4	89.0	6.4%	6.0	3.2%*	83.0	6.9%*		
e2/e2	7.0	0.5%	2.0	1.1%	5.0	0.4%		
APOE-e4 allele degree							**8.13**	**0.017**
No e4 allele	861.0	62.1%	110.0	57.9%	751.0	62.7%		
One copy of e4 allele	437.0	31.5%	74.0	38.9%*	363.0	30.3%*		
Two copies of e4 allele	89.0	6.4%	6.0	3.2%	83.0	6.9%		
Sleep Apnea	255.0	18.4%	24.0	12.6%	231.0	19.3%	**4.86**	**0.028**
		*n=203*		*n=39*		*n=164*		
Hippocampal Volume *mean (SD)*	6.5	(0.9)	6.3	(0.8)	6.6	(0.98)	**2.04**	**0.023**
		*n=200*		*n=39*		*n=161*		
WMH Volume *mean (SD)*	7.2	(21.8)	8.3	(33.8)	6.9	(17.84)	−0.24	0.406
		*n=304*		*n=13*		*n=291*		
Abnormal Amyloid levels	135.0	44.4%	5.0	38.5%	130.0	44.7%	0.19	0.659
		*n=1266*		*n=188*		*n=1078*		
MOCA Total Score	23.9	(5.1)	23.2	(4.8)	24.3	(5.15)	**2.23**	**0.013**

Data represented as n, % unless otherwise specified. *Indicates statistically different groups based on post hoc analyses. Individual variable sample sizes listed for those with more than 10% of sample missing data. WMH = White Matter Hyperintensities. Statistically significant associations bolded.

**Table 2:** Sample characteristics by OSA status and race.

	Black participants					White participants						
	OSA+(*n*=24)		OSA-(*n*=166)		*F/t/X^2^*	Sig	OSA+(*n*=230)	OSA-(*n*=962)			*F/t/X^2^*	Sig
Age *mean(SD)*	69.1	(7.5)	68.4	8.6	−0.39	0.348	70.36	(7.6)	69.83	(8.4)	−0.88	0.190
Female sex	15	62.5%	125	75.3%	1.77	0.216	**100**	**43.3%**	**573**	**59.3%**	**19.45**	**<0.001**
*Education*												
Years {mean±SD}	14.83	(2.8)	15.56	(2.8)	1.19	0.118	16.01	(2.6)	16.21	(2.8)	0.97	0.165
<9 | Elementary School	0	0.0%	1	0.6%	1.07	0.784	2	0.9%	16	1.7%	4.37	0.224
9-12 | High School	6	25.0%	32	19.3%			23	10%	104	10.8%		
13-16 | College	11	45.8%	69	41.6%			115	50%	412	42.8%		
>17 | Graduate School	7	29.2%	64	38.6%			90	39.1%	430	44.7%		
*Cognitive Status*												
Normal Cognitive Status	13	54.2%	93	56.0%	1.65	0.648	100	43.3%	465	48.1%	4.12	0.249
Impaired, not MCI	1	4.2%	10	6.0%			8	3.5%	32	3.3%		
MCI	9	37.5%	46	27.7%			89	38.5%	306	31.7%		
Dementia	1	4.2%	17	10.2%			34	14.7%	163	16.9%		
*APOE-e4*												
APOE e4 Carrier	9	37.5%	71	42.8%	0.24	0.625	81	35.1%	365	37.8%	0.60	0.442
Frequency of alleles												
e3/e3	11	45.8%	65	39.2%	1.28	0.937	127	55.0%	516	53.4%	2.73	0.741
e3/e4	8	33.3%	62	37.3%			59	25.5%	280	29.0%		
e3/e2	4	16.7%	28	16.9%			21	9.1%	82	8.5%		
e2/e4	1	4.2%	5	3.0%			18	7.8%	65	6.7%		
e4/e4	0	0.0%	4	2.4%			4	1.7%	20	2.1%		
e2/e2	0	0.0%	2	1.2%			2	0.9%	3	0.3%		
APOE-e4 allele degree					0.41	0.814					1.40	0.496
No e4 allele	15	62.5%	95	57.2%			150	64.9%	601	62.2%		
One copy of e4 allele	8	33.3%	66	39.8%			63	27.3%	300	31.1%		
Two copies of e4 allele	1	4.2%	5	3.0%			18	7.8%	65	6.7%		
		*n=5*		*n=34*				*n=35*		*n=129*		
Hippocampal Volume *mean (SD)*	6.3	(1.1)	6.3	(0.8)	−0.02	0.493	6.5	(1.0)	6.6	(0.9)	0.99	0.162
		*n=5*		*n=34*				*n=35*		*n=126*		
WMH Volume *mean (SD)*	8.1	(14.5)	8.31	(35.9)	0.01	0.494	6.4	(9.6)	7.1	(19.6)	0.21	0.415
		*n=0*		*n=13*				*n=13*		*n=291*		
Abnormal Amyloid levels	0	0.0%	13	100%	-	-	**15**	**27.3%**	**115**	**48.7%**	**8.31**	**0.004**
		*n=24*		*n=164*				*n=210*		*n=868*		
MOCA Total Score	24.2	(4.1)	23.0	(4.9)	−1.14	0.129	24.0	(4.6)	24.0	(5.3)	0.02	0.492

Data represented as n, % unless otherwise specified. *Indicates statistically different groups based on post hoc analyses. Individual variable sample sizes listed for those with more than 10% of sample missing data. WMH = White Matter Hyperintensities. Statistically significant associations bolded.

### Main effects in the total sample

Logistic Regression analyses were carried out in the total sample to assess the effects of sleep apnea and APOE-e4 on the likelihood of having abnormal amyloid levels. The overall model was statistically significant, (*χ*^2^_(5)_ = 68.433, *p* < 0.001) explained 27% of the variation in abnormal amyloid levels (Nagelkerke *R*^2^) and correctly predicted 70.7% of cases. Both sleep apnea (*p* = 0.014) and APOE-e4 (*p* < 0.001), but not their interaction, were significant. Contrast analyses revealed that odds of abnormal amyloid levels with one e4 allele were 4.53 times those of having no e4 allele (*p* < 0.001), and odds of abnormal amyloid levels with two e-4 alleles were 30.90 times those of having no e4 allele (*p* = 0.001). In subsequent analyses, APOE-e4, but not sleep apnea or their interaction was associated with a higher likelihood of having mild cognitive impairment or dementia (*χ*^2^_(5)_ = 44.183, *p* < 0.001), with being an APOE-e4 carrier, regardless of the number of alleles being more associated than not being a carrier (*p* < 0.001; see Table 3 for results of the fully adjusted models).

**Table 3:** Logistic regression analyses of the influence of APOE-e4, OSA, and their interaction on amyloid beta and cognitive status.

Variables	*β*	S.E.	OR	(95% CI)	Sig.	*β*	S.E.	OR	(95% CI)	Sig.	*β*	S.E.	OR	(95% CI)	Sig.
Abnormal Amyloid Levels															
	Total sample (*n* = 301)					Black participants (*n* = 13)					White participants (*n* = 288)				
Race	−0.29	0.65	0.75	0.21–2.67	0.659	–	–	–	–	–	–	–	–	–	–
Sex	0.04	0.28	1.05	0.60–1.81	0.876	–	–	–	–	–	0.07	0.29	1.07	0.61–1.89	0.808
Age	0.04	0.02	1.04	1.01–1.08	**0.026**	–	–	–	–	–	0.04	0.02	1.04	0.99–1.07	0.060
Education	–0.05	0.05	0.95	0.87–1.04	0.274	–	–	–	–	–	−0.06	0.05	0.95	0.86–1.04	0.236
APOE-e4_1_	1.58	0.30	4.84	2.66–8.78	**<0.001**	–	–	–	–	–	1.63	0.32	5.09	2.75–9.45	**<0.001**
APOE-e4_2_	3.68	1.01	38.73	4.90–31.22	**<0.001**						3.66	1.05	38.75	4.89–306.57	**<0.001**
OSA	–1.54	0.64	0.22	0.06–0.75	**0.016**						–1.52	0.64	0.22	0.06–0.76	**0.017**
OSA*APOE-e4	0.56	0.82	1.74	0.35–8.62	0.496						0.49	0.82	1.64	0.33–8.16	0.548
Normal cognitive status															
	*n* = 1382					*n* = 190					*n* = 1192				
Race	0.31	0.17	1.36	0.98–1.90	0.066	–	–	–	–	–	–	–	–	–	–
Sex	0.55	0.12	1.74	1.37–2.19	**<0.001**	0.74	0.38	2.04	0.98–4.32	0.055	0.58	0.23	1.79	1.39–2.30	**<0.001**
Age	–0.05	0.01	0.95	0.94–0.96	**<0.001**	–0.16	0.02	0.89	0.85–0.93	**<0.001**	–0.05	0.01	0.96	0.94–0.97	**<0.001**
Education	0.13	0.02	1.14	1.09–1.19	**<0.001**	0.04	0.06	1.05	0.93–1.18	0.459	0.15	0.02	1.16	1.11–1.22	**<0.001**
APOE-e4_1_	–0.67	0.14	0.51	0.39–0.67	**<0.001**	–0.10	0.36	0.90	0.45–1.83	0.779	–0.76	0.15	0.47	0.5–0.63	**<0.001**
APOE-e4_2_	–1.14	0.28	0.32	0.19–0.55	**<0.001**	–0.72	1.05	0.49	0.06–3.78	0.491	–1.21	0.29	0.30	0.17–0.52	**<0.001**
OSA	–0.04	0.18	0.96	0.67–1.37	0.829	0.09	0.61	1.09	0.33–3.63	0.887	–0.07	0.19	0.93	0.64–1.35	0.696
OSA*APOE-e4	0.03	0.33	1.03	0.54–1.99	0.926	0.13	1.03	1.14	0.15–8.54	0.898	0.08	0.36	1.08	0.54–2.18	0.826

A series of logistic regressions were performed in the total and stratified samples with data for all variables. At the intersection of APOE-e4 and OSA only 13 Black participants had data, therefore analyses not run in this group. Reference groups: No e4 alleles, White, Male, No OSA. Statistically significant associations bolded.

OSA, Obstructive Sleep Apnea; APOE-e4_1_, One e4 allele; APOE-e4_2_, Two e4 alleles.

Analysis *via* two-way ANOVA revealed that both sleep apnea (*F*_1,199_ = 6.058, *p* = 0.015) and APOE-e4 (*F*_2,198_ = 5.459, *p* = 0.005) independently, and their interaction (*F*_2,198_ = 3.827, *p* = 0.23), were significantly associated with WMH volume. Tukey’s multiple comparison test revealed that having one allele (*p* = 0.020) had a greater association with WMH volume than both not being a carrier and having two alleles (not significant). Additionally, APOE-e4 (*F*_1,1,268_ = 12.324, *p* < 0.001) independently and the interaction of APOE-e4 and sleep apnea (*F*_1,1,268_ = 3.135, *p* = 0.044), but not sleep apnea independently were associated with performance on the MOCA. Tukey’s multiple comparison test showed that, being an APOE-e4 carrier, regardless of the number of alleles had a greater association with MOCA than not being a carrier (*p* < 0.001), though no difference was found in performance in those with one compared to two alleles (see Table 4 for results of the fully adjusted models).

**Table 4:** Two-way ANOVA analyses of the effects of APOE-e4 and OSA on Hippocampal & White Matter hyperintensity volume, and MoCA performance.

Variables	SS	df	MS	*F*	Sig.	SS	df	MS	*F*	Sig.	SS	df	MS	*F*	Sig.
Hippocampal Volume															
	Total sample (*n* = 200)					Black participants (*n* = 39)					White participants (*n* = 161)				
Race	2.12	1	2.12	3.43	0.066	–	–	–	–	–	–	–	–	–	–
Sex	16.10	1	16.10	26.06	**<0.001**	1.14	1	1.14	2.51	0.124	12.97	1	12.97	19.71	**<0.001**
Age	34.77	1	34.77	56.26	**<0.001**	0.90	1	0.90	1.98	0.169	33.12	1	33.12	50.32	**<0.001**
Education	0.65	1	0.65	1.06	0.306	0.08	1	0.08	0.18	0.677	0.85	1	0.85	1.29	0.258
OSA	1.17	1	1.17	1.89	0.171	0.68	1	0.68	1.51	0.229	0.98	1	0.98	1.49	0.225
OSA*APOE-e4	1.76	2	0.88	1.42	0.244	2.33	2	1.17	2.57	0.094	2.01	2	1.01	1.53	0.221
APOE-e4	1.81	2	0.90	1.46	0.234	0.49	2	0.24	0.54	0.591	2.33	2	1.17	1.77	0.174
Contrast e4_0_–e4_1_					0.133					0.382					0.090
Contrast e4_0_–e4_2_					0.261					0.439					0.263
Contrast e4_1_–e4_2_					0.979					0.999					0.886
White Matter Hyperintensities															
	*n* = 197					*n* = 39					*n* = 158				
Race	64.07	1	64.07	0.15	0.702	–	–	–	–	–	–	–	–	–	–
Sex	3997.80	1	3997.80	9.17	**0.003**	177.24	1	177.24	6.05	**0.020**	1726.15	1	1726.15	5.46	**0.021**
Age	1071.42	1	1071.42	2.46	0.119	113.18	1	113.18	3.86	0.059	542.47	1	542.47	1.72	0.192
Education	245.97	1	245.97	0.56	0.453	22.22	1	22.22	0.76	0.391	51.92	1	51.92	0.16	0.686
OSA	3402.71	1	3402.71	7.81	**0.006**	11038.39	1	11038.39	376.45	**<0.001**	63.38	1	63.38	0.20	0.655
OSA*APOE-e4	3537.65	2	1768.83	4.06	**0.019**	15752.61	2	7876.30	268.61	**<0.001**	1216.28	2	608.14	1.92	0.150
APOE-e4	4685.64	2	2342.82	5.38	**0.005**	18377.67	2	9188.84	313.38	**<0.001**	59.05	2	29.52	0.09	0.911
Contrast e4_0_–e4_1_					0.936					0.079					0.667
Contrast e4_0_–e4_2_					**0.002**					**<0.001**					0.911
Contrast e4_1_–e4_2_					**0.004**					**<0.001**					0.850
MoCA Score															
	*n* = 1266					*n* = 188					*n* = 1078				
Race	99.20	1	99.20	4.37	**0.037**										
Sex	315.44	1	315.44	13.91	**<0.001**	78.94	1	78.94	4.34	**0.039**	272.77	1	272.77	11.71	**<0.001**
Age	1653.29	1	1653.29	72.89	**<0.001**	734.93	1	734.93	40.42	**<0.001**	1058.70	1	1058.70	45.46	**<0.001**
Education	2372.17	1	2372.17	104.58	**<0.001**	248.14	1	248.14	13.65	**<0.001**	2105.54	1	2105.54	90.40	**<0.001**
OSA	2.60	1	2.60	0.11	0.735	7.72	1	7.72	0.42	0.516	5.83	1	5.83	0.25	0.617
OSA*APOE-e4	117.86	2	58.93	2.60	0.075	17.19	2	8.60	0.47	0.624	91.17	2	45.59	1.96	0.142
APOE-e4	657.27	2	328.63	14.49	**<0.001**	3.31	2	1.65	0.09	0.913	732.90	2	366.45	15.73	**<0.001**
Contrast e4_0_–e4_1_					**0.002**					0.739					**0.001**
Contrast e4_0_–e4_2_					**<0.001**					0.756					**<0.001**
Contrast e4_1_–e4_2_					**0.004**					0.867					**0.004**

A series of two-way ANOVAs were performed in the total and stratified samples with data for all variables. Contrast analyses demonstrated influence of APOE-e4 allele dosage. Reference groups: No e4 alleles, White, Male, No OSA. OSA, Obstructive Sleep; e4_0_ = No e4 allelese4_1_ = One e4 allele; e4_2_ = Two e4 alleles. Statistically significant associations bolded.

### Main effects in Black/African American and white groups

Fully adjusted models exploring the influence of APOE-e4, OSA, and their interaction on abnormal levels of amyloid while controlling for race, sex, age, and years of education indicated that only age also demonstrated a relationship with abnormal levels of amyloid, and the continued significance of the model suggested that race, sex, years of education, and to a lesser degree age, had no influence on the associations explored (see Table 3 for results of the fully adjusted models). However, given the disparate sample sizes across race, we ran subsequent analyses in Black/African American and White participant groups separately to ensure statistical rigor when examining possible group differences.

Following grouping of the dataset into separate cohorts of Black/African America and White participants, Logistic regression analyses in each group separately, revealed that in White participants, both sleep apnea (*p* = 0.022) and APOE-e4 (*p* < 0.001), but not their interaction, still predicted the presence of abnormal amyloid levels (*χ*^2^_(5)_ = 68.433, *p* < 0.001) Contrast analyses revealed that odds of abnormal amyloid levels with one e4 allele were 5.22 times those of having no e4 allele, and odds of abnormal amyloid levels with two e-4 alleles were 42.78 times those of having no e4 allele (*p* < 0.001). These analyses were not run for Black/African American participants because at the intersection of APOE-e4 and abnormal amyloid only 13 Black/African American participants had data. Also, in White, but not Black/African American participants, APOE-e4, but not sleep apnea or their interaction continued to be associated with a higher likelihood of having mild cognitive impairment or dementia (*χ*^2^_(5)_ = 45.663, *p* < 0.001), with being an APOE-e4 carrier, regardless of the number of alleles being more associated than not being a carrier (*p* < 0.001).

In separate group analyses with Two-way ANOVA white, but not Black/African American participants, continued to demonstrate an association between performance on the MOCA and APOE-e4 (*F*_1,1,080_ = 14.003, *p* < 0.001) independently but not sleep apnea independently or the interaction of APOE-e4 and sleep apnea. Tukey’s multiple comparison test continued to show that being an APOE-e4 carrier, regardless of the number of alleles had a greater association with MOCA than not being a carrier (*p* < 0.001), though no difference was found in performance in those with one compared to two alleles. Analysis *via* two-way ANOVA also revealed that, Black/African American but not White participants continued to demonstrate a significant association between WMH volume and both sleep apnea (*F*_1,39_ = 229.248, *p* < 0.001) and APOE-e4 (*F*_2,38_ = 246.115, *p* < 0.001) independently, and their interaction (*F*_2,38_ = 274.095, *p* < 0.001). *Post hoc* analyses indicated that having two e-4 alleles had a greater association with WMH volume than both having one e-4 allele and not being carrier (*p* < 0.001), though no difference was shown between not being a carrier and having one e-4 allele. Additionally, in Black/African American but not White participants, an association emerged between Hippocampal volume and sleep apnea independently (*F*_1,38_ = 4.274, *p* = 0.047) and the interaction between sleep apnea and APOE-e4 (*F*_2,37_ = 7.771, *p* = 0.002), though the association with APOE-e4 independently only trended toward significance (*F*_2,37_ = 2.476, *p* = 0.061).

## Discussion

In the present study we utilized the NACC UDS to explore the combined influence of OSA and the presence of APOE-e4 allele on biomarkers of AD, and any differences across Black/African American and White samples. Our results regarding the independent relationships of the presence of OSA and APOE-e4 alleles mirror what the literature suggests (Roses, 1996; Tardiff et al., 1997; Gottlieb et al., 2004; Uyrum et al., 2015; Ju et al., 2016; Liguori et al., 2017; Elias et al., 2018; Bubu et al., 2019, 2020). Specifically, in the total analytic sample [total sample (amyloid, *n* = 301), WMH, *n* = 197, MOCA, *n* = 1,266] the presence of OSA was independently associated with abnormal amyloid, and the presence of APOE-e4 alleles were associated with abnormal amyloid, as well as WMH volume, while only APOE-e4 was independently associated with performance on the MOCA and cognitive status in this sample. The only significant interactions between the presence of sleep apnea and the presence of APOE-e4 alleles were relative to WMH volume in the total sample and in both WMH and Hippocampal volume in Black/African American participants. However, we must acknowledge the possibility that relationships with some of the AD biomarkers explored may be obscured due to data availability for amyloid metrics overall in the NACC UDS repository.

Research indicates that APOE-e4 is the gene with the strongest impact on risk of late-onset Alzheimer’s. Though research shows that a higher percentage of Black/African Americans have at least one copy of the e4 allele, it also indicates that having the e4 allele has a null to weaker association with increased AD risk for this group (Qian et al., 2017; Rajan et al., 2019; Anonymous, 2021; Beydoun et al., 2021; Weiss et al., 2021). While our results do demonstrate a higher proportion of Black/African Americans having at least one APOE-e4 allele (44.4% vs. 40.4%), they also demonstrate that the presence of an APOE-e4 allele was associated with WMH and Hippocampal volume, but not cognitive status in Black/African Americans in our sample. These results add to the literature that posits differential associations with WMH being the driver of disparate WMH burden seen by race (Seixas et al., 2021), especially since no significant differences were seen in WMH burden across race in this sample. However, given the fact that we did not conduct analyses subdivided by cognitive status, caution must be taken with inferences related to the results surrounding cognitive status. Additionally, though research indicates Black/African American individuals are more likely to have sleep apnea (Dudley and Patel, 2016), there was no difference in the proportion of sleep apnea across Black/African American and white samples.

The continued significance of the model in analyses controlling for race, sex, age, and years of education suggested that these variables, had no influence on the associations explored, however, to ensure statistical rigor, especially given the disparate sample sizes across race, sleep apnea and amyloid status, we ran subsequent analyses in Black/African American and White participant groups separately. These analyses revealed that in White participants, both sleep apnea and APOE-e4 but not their interaction, still predicted the presence of abnormal amyloid levels, and for both performance on the MOCA and cognitive status there was still an association with APOE-e4 independently but not sleep apnea independently or the interaction of APOE-e4 and sleep apnea. Additionally, in Black/African American but not White participants there was an association between WMH volume and both sleep apnea and APOE-e4 independently, and their interaction, while a previously unseen association emerged between Hippocampal volume and sleep apnea independently and the interaction between sleep apnea and APOE-e4, though not APOE-e4 independently. Though we must take care to insure interpretation of these results in accordance with the aforementioned discrepancies in CSF amyloid response rates. These results attest to the importance of statistical measurement when approaching these analyses. Our covariate controlled analyses indicated a relationship between sleep apnea and APOE-e4 alleles with amyloid metrics without the influence of race, however, what it was not able to demonstrate was that the lack of influence may have been because Black/African American participants were likely not represented in the analyses (Jean-Louis et al., 2020).

As expected, given the results of the analyses in sleep apnea and APOE-e4 separately, there were few significant interactions between the presence of sleep apnea and APOE-e4 relative to the biological and clinical markers of AD explored in White and Black/African American participants. However, analyses did reveal that the interaction of APOE-e4 and the presence of sleep apnea were significantly associated with WMH and hippocampal volume in Black/African American, but not White participants. Though the database may not have been powered enough to fully elucidate the combined influence of OSA and the presence of APOE-e4 allele on biomarkers of AD across Black/African American and White participants, the results of these analyses indicate that there is a possibility, that it may exert different influences in different populations, and galvanizes the need for further research.

It is important to acknowledge that race is a social constructed concept, a malleable and heterogenous social category created through time, state, and social interactions (Rajan et al., 2017; Anonymous, 2021). Historically, the notion of race as a biological determinant has been supported by false narratives and inaccurate ideologies that there are inherent biological differences between racial and ethnic groups. It is vital to explore these racial and ethnic differences so that scientists, researchers, and physicians do not reinforce the systemic inequalities and health disparities among the most vulnerable disadvantage patients. In that vein, our study further demonstrates the strong need for engaging, recruiting, and retaining diverse populations in research and clinical trials. It is well established that Black/African American populations are at an increased risk of developing AD and may face limited access to healthcare (Rajan et al., 2017; Anonymous, 2021), which could serve to concatenate into increased vulnerability in these groups. Further studies will need to be done to elucidate the role of sleep disruption and genetic factors on AD progression, especially in the context of race/ethnicity, and other social demographics. Though our hypothesis that the interaction/combined effects of modifiable and fixed risk factors may explain some of the increased risk seen in Black/African American populations were not supported; it was likely due to power in specific variables. This coupled with the results of our exploratory analyses suggest not only the need for continued exploration of the interaction/combined effects of modifiable and fixed risk factors, but also for future studies to explore the cardiovascular influence and relationship of white matter hyperintensities in AD risk for Black/African Americans.

There are several limitations to this study. First, the presence or absence of sleep apnea was determined within the clinical interview, but is derived from self-report to the clinician, which introduces the possibility for recall bias and subjective interpretation of sleep symptoms. Secondly, though the study population is relatively large, all variables are not represented equivalently. While our ability to analyze some parameters of our hypotheses were hindered by this discrepancy in sample size across variables, the rigorous approach necessitated by the discrepancy also allowed for a demonstration that controlling for race is not always an adequate way of dealing with the potential influence of racial/ethnic differences. The variable that was most disparate was abnormal levels of the AD biomarker Aβ. Thus, we utilized all available assessments of abnormal amyloid (presence of abnormal amyloid on PET scan, presence of abnormal amyloid in CSF, and amyloid positivity status based on CSF amyloid levels) in assessments to ensure reasonable levels of inclusion. Though it is unfortunate that Aβ metrics were the ones with the least amount of data, especially in Black/African American participants, it is unsurprising given that recruitment and retention research indicates the invasive nature of procedures like lumbar puncture as a barrier for Black/African American inclusion (Howell et al., 2016; Blazel et al., 2020). Therefore, these disparate numbers may reflect a need to shift to less invasive measures, like plasma biomarker analyses to augment Black/African American participation in future studies. Finally, the cross-sectional nature of the study precludes the identification of any causal links between sleep, APOE-e4, and AD, and longitudinal analyses, that also allow for examining the associations of cognitive status, should be conducted to substantiate these findings.

## Conclusion

In summary, our results confirm that sleep apnea as a modifiable risk factor and APOE-e4 alleles as a genetic risk factor are each independently associated with abnormal levels of amyloid, and WHM volumes. We further demonstrate that both sleep apnea and APOE-e4 are interactively associated with WHM with race-stratified analyses showing that this sleep apnea-APOE4 interaction on WHM occurred only in Black participants. These findings, bolster the need for further research exploring the interaction/combined effects of modifiable and fixed risk factors for AD, especially in Black/African American populations, where this interaction may partially explicate increased levels of risk.

## Data availability statement

The data analyzed in this study is subject to the following licenses/restrictions: Data must be requested, and is protected by a data use agreement ensuring use of the data solely by the individuals identified in the data request. Requests to access these datasets should be directed to https://naccdata.org/requesting-data/data-request-process.

## Ethics statement

Ethical review and approval was not required for the study on human participants in accordance with the local legislation and institutional requirements. The patients/participants provided their written informed consent to participate in this study.

## Author contributions

AT conceptualized and designed the study, contributed to the development of the scientific arguments, and prepared tables and figures. CL contributed to the development of the scientific arguments, processed, and analyzed the data and contributed to data interpretation. AB and DO contributed to the discussion of the scientific arguments and reviewed/edited the manuscript. OB and AS reviewed and edited the manuscript. All authors have read and agreed to the published version of the manuscript.

## Funding

The NACC database is funded by NIA/NIH Grant U24 AG072122. NACC data are contributed by the NIA-funded ADRCs: P30 AG062429 (PI James Brewer, MD, PhD), P30 AG066468 (PI Oscar Lopez, MD), P30 AG062421 (PI Bradley Hyman, MD, PhD), P30 AG066509 (PI Thomas Grabowski, MD), P30 AG066514 (PI Mary Sano, PhD), P30 AG066530 (PI Helena Chui, MD), P30 AG066507 (PI Marilyn Albert, PhD), P30 AG066444 (PI John Morris, MD), P30 AG066518 (PI Jeffrey Kaye, MD), P30 AG066512 (PI Thomas Wisniewski, MD), P30 AG066462 (PI Scott Small, MD), P30 AG072979 (PI David Wolk, MD), P30 AG072972 (PI Charles DeCarli, MD), P30 AG072976 (PI Andrew Saykin, PsyD), P30 AG072975 (PI David Bennett, MD), P30 AG072978 (PI Neil Kowall, MD), P30 AG072977 (PI Robert Vassar, PhD), P30 AG066519 (PI Frank LaFerla, PhD), P30 AG062677 (PI Ronald Petersen, MD, PhD), P30 AG079280 (PI Eric Reiman, MD), P30 AG062422 (PI Gil Rabinovici, MD), P30 AG066511 (PI Allan Levey, MD, PhD), P30 AG072946 (PI Linda Van Eldik, PhD), P30 AG062715 (PI Sanjay Asthana, MD, FRCP), P30 AG072973 (PI Russell Swerdlow, MD), P30 AG066506 (PI Todd Golde, MD, PhD), P30 AG066508 (PI Stephen Strittmatter, MD, PhD), P30 AG066515 (PI Victor Henderson, MD, MS), P30 AG072947 (PI Suzanne Craft, PhD), P30 AG072931 (PI Henry Paulson, MD, PhD), P30 AG066546 (PI Sudha Seshadri, MD), P20 AG068024 (PI Erik Roberson, MD, PhD), P20 AG068053 (PI Justin Miller, PhD), P20 AG068077 (PI Gary Rosenberg, MD), P20 AG068082 (PI Angela Jefferson, PhD), P30 AG072958 (PI Heather Whitson, MD), P30 AG072959 (PI James Leverenz, MD). The authors of this work are supported by funding from the National Institutes of Health: Authors from the Center for Translational Sleep & Circadian Sciences and the Media and Innovation Lab at the University of Miami are supported by R01AG067523 and R01HL142066; In addition, AT is supported by R25HL10544; In addition, AS is supported by R01HL152453 and R01AG056531. AB is supported by the following NIH grant 3P30AG066512-03S2. OB is supported by the following NIA/NIH grant K23AG068534, Alzheimer’s Association [AARG-D- 21-848397] and BrightFocus Foundation [A2022033S]. The funding sources had no role in the design, conduct, or analysis of the study, or in the submission decision.

## Conflict of interest

The authors declare that the research was conducted in the absence of any commercial or financial relationships that could be construed as a potential conflict of interest.

The handling editor declared a shared affiliation with the authors AB and OB at the time of review.

## Publisher’s note

All claims expressed in this article are solely those of the authors and do not necessarily represent those of their affiliated organizations, or those of the publisher, the editors and the reviewers. Any product that may be evaluated in this article, or claim that may be made by its manufacturer, is not guaranteed or endorsed by the publisher.

## Footnotes

^1^ http://idealab.ucdavis.edu/
